# New Appliances and Things Medical

**Published:** 1893-07-29

**Authors:** 


### NEW APPLIANCES AND THINGS MEDICAL.

[All preparations, appliances, novelties, etc., of which a notice is desired, should be sent for the Editor, to care of The Manager, 428, Strand, London, W.C.]

### WATERPROOF SHEETING.

In all our hospitals and charitable institutions waterproof sheeting is in constant use, and being subject to continual wear and tear, neither time nor trouble is lost in securing a really durable article. Messrs. Anderson, of 37, Queen Victoria Street, E.C., are justly renowned for the excellence of their indiarubber goods. Their waterproof sheeting is of excellent quality; it is durable, soft, and pliable. Confident in the superiority of this speciality, the makers offer to supply a specimen sheet to any institution not already using their sheeting. We can confidently recommend hospitals to take advantage of this offer, as they will thus have an opportunity of testing the excellence of Messrs. Anderson's sheeting.

---

### CARBO-EUCALYPTINE VOLATILE SANITARY TABLETS.

(The Eureka Manufacturing Company, 146, Chatsworth Road, Clapton Park, N.E.)

These are of two forms, one for hanging on the wall, the other by suitable wire attachments for placing in the pans of w.c.'s, urinals, sinks, dust holes, &c. The active material consists of a crystalline volatile coal-tar derivative combined with eucalyptus. They have been well tested and are so much approved of that the Houses of Parliament and many hospitals and hotels make use of them. Again, they have been found of considerable value in cholera camps, and we think that for the purpose for which they are designed one can find nothing more efficacious. Their use by the persons in authority at the institutions mentioned is quite sufficient evidence of their value. Personally we have tried them in a stable, over a foul drain, and in an ordinary urinal basin, each time with success.

---

### "SANITAS."

The disinfecting Sanitas preparations are too widely known to need introduction, but the vague ideas of what constituted an efficient disinfectant have given place to tests which bacteriology has rendered absolutely conclusive, and by which the claims of all so-called disinfectants must be judged. This the "Sanitas" Company have recognised, and the elaborate experiments of Dr. A. B. Griffiths, a well-known bacteriologist, have demonstrated that Sanitas oil and fluid are preparations upon which we may place every reliance as accomplishing all that disinfectant and germicidal agents can do.

---

### "FRANZ JOSEF" NATURAL PURGATIVE WATER.

A considerable experience of the value of this water as a saline aperient enables us to highly commend it as one of the pleasantest, most reliable, and regular in strength and quality in the market. It will rapidly gain favour with the medical profession as it becomes more widely known.

